# Effect of* Kangfuxin* Solution on Chemo/Radiotherapy-Induced Mucositis in Nasopharyngeal Carcinoma Patients: A Multicenter, Prospective Randomized Phase III Clinical Study

**DOI:** 10.1155/2016/8692343

**Published:** 2016-06-07

**Authors:** Yangkun Luo, Mei Feng, Zixuan Fan, Xiaodong Zhu, Feng Jin, Rongqing Li, Jingbo Wu, Xia Yang, Qinghua Jiang, Hongfang Bai, Yecai Huang, Jinyi Lang

**Affiliations:** ^1^Department of Radiation Oncology, Sichuan Cancer Hospital, Chengdu 610041, China; ^2^Department of Radiation Oncology, Affiliated Cancer Hospital of Guangxi Medical University, Nanning 530021, China; ^3^Department of Oncology, Guizhou Cancer Hospital, Affiliated Hospital of Guiyang Medical College, Guiyang 550003, China; ^4^Department of Radiation Oncology, First Affiliated Hospital of Kunming Medical University, Kunming 650032, China; ^5^Department of Oncology, Affiliated Hospital of Sichuan Medical University, Luzhou 646000, China; ^6^Kelun Pharmaceutical Research Institute, Sichuan Kelun Pharmaceutical Co., Ltd., Chengdu 610071, China

## Abstract

*Objective*. To evaluate the efficacy and safety of* Kangfuxin* Solution, a pure Chinese herbal medicine, on mucositis induced by chemoradiotherapy in nasopharyngeal carcinoma patients.* Methods*. A randomized, parallel-group, multicenter clinical study was performed. A total of 240 patients were randomized to receive either* Kangfuxin* Solution (test group) or compound borax gargle (control group) during chemoradiotherapy. Oral mucositis, upper gastrointestinal mucositis, and oral pain were evaluated by Common Terminology Criteria for Adverse Events (CTCAE) v3.0 and the Verbal Rating Scale (VRS).* Results*. Of 240 patients enrolled, 215 were eligible for efficacy analysis. Compared with the control group, the incidence and severity of oral mucositis in the test group were significantly reduced (*P* = 0.01). The time to different grade of oral mucositis occurrence (grade 1, 2, or 3) was longer in test group (*P* < 0.01), and the accumulated radiation dose was also higher in test group comparing to the control group (*P* < 0.05). The test group showed lower incidence of oral pain and gastrointestinal mucositis than the control group (*P* < 0.01). No significant adverse events were observed.* Conclusion*.* Kangfuxin* Solution demonstrated its superiority to compound borax gargle on mucositis induced by chemoradiotherapy. Its safety is acceptable for clinical application.

## 1. Introduction

Mucositis refers to secondary mucosal damage in the oral cavity, pharynx, larynx, esophagus, or other parts of the gastrointestinal tract. It normally occurs during cancer treatment. Almost all head and neck cancer patients who receive radiotherapy experience mucositis [1]. Oral mucositis usually starts with mucosal inflammation, characterized by erythema, and fused ulcers [2]. The main clinical symptom includes pain that affects normal eating, with secondary effects such as dehydration, dysgeusia, and malnutrition. Patients with bone marrow suppression may manifest secondary infection that leads to mucositis [3]. Gastrointestinal mucositis is characterized by pain, nausea, vomiting, and diarrhea [4]. On the one hand, severe mucositis leads to chemotherapy dose reduction or radiotherapy interruption, which affects the prognosis [5, 6]. On the other hand, it also leads to a lower quality of life, weight loss, and prolonged hospitalization as well as additional analgesic treatment, parenteral nutrition, liquid replacement therapy, and drugs for the treatment and prevention of serious infections, which increase the economic burden of patients [7–10].

Nasopharyngeal carcinoma (NPC) is one of the most common head and neck cancers in South China [11]. Chemoradiotherapy is the standard treatment. The incidence of mucositis is 100% in patients receiving radiotherapy [12]. The main symptom is pain, and the pain grade gradually rises with increasing radiation dose [13]. Since oral mucositis caused by chemoradiotherapy is “easy to diagnose, difficult to handle,” several drugs aimed at preventing and treating mucositis have been reported in recent years. However, drugs with clear efficacy for clinical recommendation have yet to be developed or discovered [14].* Kangfuxin* Solution is a pure Chinese herbal medicine that is extracted from the American cockroach. It has been shown that* Kangfuxin* Solution may reduce the pain and discomfort of patients after chemoradiotherapy by inhibiting radiation damage-induced opening of the calcium-dependent potassium channel, thus maintaining normal cell function [15]. It is also believed to improve neutrophil muscle actin function and increase the number of neutrophils after radiation injury [16]. In addition, it promotes the synthesis and secretion of extracellular matrix in the wound site [17], thus improving the healing of mucositis caused by radiation. However, previous studies were limited by poor study design or small sample sizes and were not prospective. Therefore, the current study used a multicenter, randomized, parallel-group trial design to evaluate the efficacy and safety of* Kangfuxin* Solution in chemoradiotherapy-induced mucositis in nasopharyngeal carcinoma patients.

## 2. Materials and Methods

### 2.1. Study Patients

In this study, a multicenter, randomized, parallel-group clinical trial was performed from August 2012 to June 2014, including 240 patients with nasopharyngeal carcinoma who received treatment for the first time at one of the five hospitals including Sichuan Cancer Hospital; First Affiliated Hospital of Kunming Medical University; Guizhou Cancer Hospital, The Affiliated Cancer Hospital of Guizhou Medical University; Affiliated Cancer Hospital of Guangxi Medical University; and Affiliated Hospital of Sichuan Medical University. The study was approved by the respective hospital Ethics Committees. All the included patients provided signed informed consent. This clinical study was registered at the Chinese Clinical Trial Registry (http://www.chictr.org.cn/) with the registration number ChiCTR-IPR-15006687.

The inclusion criteria for this study were as follows: (1) histopathologically diagnosed patients without metastases; (2) patients at clinical stages I–IVB of nasopharyngeal carcinoma, according to the American Joint Committee on Cancer, 7th edition (2010); (3) patients aged 18 to 70 years, either male or female; (4) patients who underwent radical chemoradiotherapy; (5) patients with a Karnofsky score ≥70 points; and (6) patients with an expected survival of at least 6 months.

The exclusion criteria were as follows: (1) pregnant women, nursing mothers, and other female patients who were contraindicated for contraceptives during the test; (2) patients who already had active oral diseases, including oropharyngeal candidiasis and facial herpes, or other oral diseases; (3) patients with stomatitis; (4) patients with residual or recurrent nasopharyngeal carcinoma; (5) patients with severe heart, liver, kidney, blood, or nervous system or psychiatric disorders; (6) patients treated with neoadjuvant or concurrent chemotherapy with 5-fluorouracil; (7) patients treated with molecular targeted therapy; (8) diabetic patients with uncontrolled blood glucose levels; (9) patients with drug allergies, either known or suspected by a drug allergy test; (10) patients with a history of alcohol and/or drug abuse; and (11) patients who had participated in clinical trials of other drugs within the past 3 months.

### 2.2. Drugs

*Kangfuxin* Solution was manufactured by Hunan Kelun Pharmaceutical Co., Ltd. (Yueyanglou District, China) (Zhunzhi: Z43020995, 100 mL/bottle, lot number: M1204191; shelf-life: 36 months). Compound borax gargle was manufactured by Yunjia Huangpu Pharmaceutical Co., Ltd., Shanghai, China (Zhunzi H31022772, 250 mL/bottle, batch number: 120,308; shelf-life: 24 months).

### 2.3. Study Design

A multicenter, randomized, and controlled clinical trial was designed. A total of 240 subjects were randomly assigned to the test group (120 patients) or the control group (120 patients). In the test group, patients first rinsed their mouth with water before treatment to clean the oral cavity, followed by slow swallowing of 10 mL of* Kangfuxin* Solution, after gargling for 3 to 5 min with bulging cheeks alternating with sucking. In the control group, the patients first rinsed their mouth with water before treatment to clean the oral cavity, gargling with 10 mL of compound borax gargle for 3–5 min with bulging cheeks alternating with sucking, and spitting it out. Both* Kangfuxin* Solution and the compound borax gargle were administered to the patients on the first day of chemoradiotherapy, three times a day, after breakfast, lunch, and supper, respectively, until the patients were diagnosed with grade 3 oral mucositis or the patients finished the entire course of radiotherapy.

If the patients were diagnosed with grades 0–2 oral mucositis, drugs (such as* Koutai*, chlorhexidine, povidone iodine gargle, topical recombinant human epidermal growth factor, topical recombinant human basic fibroblast growth factor, topical recombinant bovine basic fibroblast growth factor, or watermelon cream) other than* Kangfuxin* Solution or compound borax gargle were used to treat oral mucositis. Hormones, antibiotics, or other treatments were not used. Any patient who received any of the above-mentioned pain control medications was excluded from the study. If the patients acquired oral fungal infections, NaHCO_3_ and antifungal agents were used for mouthwash, with proper documentation. If the patients had grade 2 pain, lidocaine was administered in the form of a mouthwash, with proper documentation.

### 2.4. Evaluation Criteria

Patients were monitored from the first day of chemotherapy or radiotherapy until the emergence of grade 3 oral mucositis. During the course of treatment, the subjects were examined for oral mucosal inflammation, including posterior pharyngeal mucosa, upper gastrointestinal mucositis, and oral pain rating using on Common Terminology Criteria for Adverse Events (CTCAE) v3.0 standards [18] and the VRS standard [19], every day from 8:00 to 10:00. The grades of oral mucositis, upper gastrointestinal mucositis, and oral pain were recorded. The radiation dose was recorded with the start and end times of radiotherapy, break time, the cumulative radiation dose, drug-related adverse reactions, and withdrawal time. The primary end-point criteria for efficacy evaluation included the incidence of oral mucositis and change of grades during radiation; the secondary end-point criteria for efficacy evaluation included the incidence of gastrointestinal mucositis and a change in the grade of oral pain during radiotherapy.

### 2.5. Chemotherapy and Radiotherapy

All patients were treated with radical radiotherapy, using intensity modulated radiation therapy, with a dose of 66–74 Gy (2.1–2.3 Gy each time) for gross tumor volume (GTV), 60–70 Gy (2.0–2.2 Gy each time) for gross tumor volume in lymph node (GTVln), 60–66 Gy (1.8–2.0 Gy each time) for clinical target volume-1 (CTV-1), 54–60 Gy (1.8–2.0 Gy each time) for clinical target volume-2 (CTV-2), and 50 Gy (1.8–2.0 Gy each time) for neck prevention. All patients received five treatments per week. The accelerator dose rate and the accuracy of the beam in all participating hospitals were based uniformly on quality assurance, which was measured and monitored to ensure the reliability of patient radiation dose during treatment.

### 2.6. Randomization and Statistical Analysis

Random codes were generated by stratified block randomization, stratified by center, using SAS software. The random numbers generated by the computer were assigned to drugs by pharmacists. The drugs were sequentially distributed to subjects according to the time of enrollment. In all participating hospitals, a smaller random number was assigned to a patient enrolled earlier. The SAS statistical package was used for statistical analysis. The rank-sum test and the *t*-test were used to evaluate the treatment for the primary end-point criteria, while the *H* chi-squared test and rank-sum test were used to evaluate the treatment for the secondary end-point criteria. *P* < 0.05 was considered statistically significant.

## 3. Results

### 3.1. Demographic Characteristics

A total of 240 patients were enrolled in this trial, including 120 in the test group and 120 in the compound borax gargle group. Twenty-five patients withdrew during the trial, and therefore data from 215 cases were eventually evaluated. The demographic data are listed in Table 1. The test and control groups were similar in terms of gender, age, height, weight, Karnofsky score, past medical history, allergies, staging, chemotherapy programs, and treatment time. The differences between the two groups were not statistically significant (*P* > 0.05).

### 3.2. Efficacy

#### 3.2.1. Incidence, Timing, and Grade of Oral Mucositis

Compared with the control group, the incidence and grade of oral mucositis were significantly lower in the test group (*P* = 0.0084; Table 2). The incidence of grade 3 oral mucositis in the test and control groups was 40.19% and 53.70%, respectively. Comparing the grades of oral mucositis in the two groups, during the trial as well as at the end of the trial, the test drug was found to reduce the severity of oral mucositis (*P* = 0.0098; Table 3).

The test group delayed the occurrence of oral mucositis. The time between start of chemoradiotherapy and occurrence of grade 1, 2, or 3 oral mucositis was significantly different between the two groups (*P* < 0.0001, *P* = 0.0014, and *P* = 0.0001, resp.; Table 4).

When the same grade of mucositis occurred, the cumulative radiation dose in the test group was significantly greater than in the control group (*P* < 0.0001, *P* = 0.0377, and *P* < 0.0001, resp.; Table 5).

#### 3.2.2. Incidence of Upper Gastrointestinal Mucositis and Oral Pain

Compared with the control group, the test group showed a reduced incidence and grade of gastrointestinal mucositis (*P* < 0.0001). Comparison of patients complaining of the highest level of oral pain between the two groups showed that the test drug reduced the incidence of high-grade oral pain. The difference between the two groups was statistically significant (*P* = 0.0003) (Table 6).

### 3.3. Safety Evaluation

A total of 108 cases (90.0%) of adverse events occurred in the test group, compared with 103 cases (85.3%) in the control group. However, using the chi-squared test for the incidence of adverse events in both groups, the difference was not statistically significant (*P* = 0.3221). Neither group experienced serious adverse events. The severity and outcome of adverse events in the two groups were not significantly different (*P* = 0.1383; *P* = 0.5732).

## 4. Discussion

Radiotherapy is the main treatment for NPC. For most patients with locoregionally advanced NPC, treatment includes chemotherapy, leading to further aggravation of oral mucositis. Almost 100% of such patients suffer from varying grades of mucositis [20]. Mucositis is considered the most painful side effect of radiotherapy, and approximately 15% of patients require hospitalization [21]. In addition, it also compromises the tolerance of normal tissue to radiation, thus limiting the radiation dose [22, 23]. Further, mucositis affects the efficacy of chemoradiotherapy and even leads to patient withdrawal. Recent studies have shown that abandonment or interruption of radiotherapy increases the number of residual tumor cells, the risk of recurrence, and metastasis, thus reducing the patient survival rate [24].

Radiation-induced mucositis involves a five-step mechanism [25]: (1) radiation first induces necrosis or apoptosis of epithelial cells and destruction of their compensation through proliferation, resulting in epithelial damage [26]; (2) severe damage is caused by the following mechanism [27]: oxidative stress, leading to cell, tissue, and vascular injury; (3) reactive oxygen may activate second messengers (such as nuclear transcription factor NF-*κ*B), proinflammatory cytokines (such as tumor necrosis factor-*α* and interleukin 6), and metabolic byproducts in the microenvironment; (4) radiation or anticancer drugs cause saliva loss, which reduces the protective effect of the mucosal surface; and (5) chemoradiotherapy reduces the number of neutrophil cells, thus decreasing immunity [28].

*Kangfuxin* Solution promotes the growth of granulation tissue and angiogenesis, accelerates shedding of necrotic tissue, and repairs all kinds of ulcers and wound surfaces. It acts as an anti-inflammatory agent by eliminating inflammatory edema. In addition, it improves the phagocytic capacity of macrophages and lymphocytes, increases serum lysozyme activity, enhances immunity, and regulates physiological equilibration and homeostasis. Studies on rats have shown that* Kangfuxin* Solution increases the number and function of neutrophils after simple trauma or radiation-induced wound. It also promotes synthesis of extracellular matrix and secretion in skin wounds caused by simple trauma or radiation damage [17]. Further, Chen et al. [16] reported that radiation-induced neutrophil actin dysfunction results in reduced neutrophil phagocytic activity as well as decreased wound neutrophil numbers and function. These effects delay healing after whole-body irradiation.* Kangfuxin* Solution restores actin function in neutrophils, thus increasing the neutrophil population in the wound. Ye et al. [29] have shown that the differences in membranous ion channel activity in peritoneal macrophages between* Kangfuxin* Solution-treated and control groups were not significant. However,* Kangfuxin* Solution activated opening of the anion channel partially in the irradiated group, reversing the inhibition after exposure to ionizing radiation [30].* Kangfuxin* Solution inhibits the opening of calcium-dependent potassium channels after radiation damage, helping to maintain normal cell function. These effects may promote wound healing [15].

According to 2013 Multinational Association for Cancer Support Treatment (MASCC)/International Association of Oral Cancer (ISOO) guidelines for secondary mucositis after cancer treatment [31], treatments for mucositis include laser therapy, cryotherapy, and drug therapy, which involve cell growth factors, anti-inflammatory drugs, antibiotics, coating agent narcotic analgesics, and natural medicine. KGF-1 is so far the only Federal Drug Administration-approved drug for the prevention of oral mucositis, but it is suitable for blood cancer [32].* Xiaoyanling* gargle can be used for prophylaxis of moderate head and neck cancer- (<50 Gy) induced oral mucositis, but it is only indicated for radiotherapy [31]. Laser therapy and cryotherapy are difficult to administer in clinical practice. Amifostine is recommended for treating gastrointestinal mucositis intravenously. Compound borax gargle contains borax, sodium bicarbonate, liquefied phenol, and glycerol. Glycerol has a protective effect on the oral mucosa. In addition, it also reacts with borax and sodium bicarbonate to produce glycerin sodium borate, which enhances the drug efficacy. Since compound borax gargle is the most commonly used gargle for treating oral mucositis, it was selected as the control drug in this study.

The study showed that the incidence of oral mucositis was significantly lower in test group (*P* = 0.0084). The incidence of grade 3 mucositis in the test and control groups was 40.19% and 53.70%, respectively. According to the definition of continuous ulcers or the pseudomembranes based on CTCAE v3.0, bleeding caused by small abrasions results in grade 3 mucositis. Patients often cannot eat orally, requiring hormone and antibiotic treatment, leading to radiotherapy interruption. Therefore,* Kangfuxin* Solution reduces the incidence of all levels of mucositis, especially high-grade mucositis, to improve patient's tolerance to radiation, ensuring the continuity of radiotherapy. These findings were consistent with previous studies. However, in contrast to previous studies, our trial used compound borax gargle as the control drug. The preventative effect of the control drug against mucositis was reported to be stronger than the methods used in the literature, such as the compound rinse oral fluid (0.9% NaCl + gentamicin + dexamethasone + Vitamin B_12_) [30], 0.2% chlorhexidine tinidazole peptide [33], or mouthwash gargle (0.9% NaCl + gentamicin + dexamethasone + lidocaine + Vitamin B) [34]. Thus,* Kangfuxin* Solution significantly reduced the incidence of mucositis, compared with the current methods commonly used clinically. In this study, grade 3 mucositis was selected as the end-point because we primarily focused on prevention. Comparing the grades of oral mucositis in the two groups, during the trial as well as at the end of the trial, the test drug also was shown to reduce the severity of oral mucositis (*P* = 0.0098). This result suggested that* Kangfuxin* Solution was effective in reducing the incidence of high-grade mucositis. Severe mucositis is normally treated with hormones and antibiotics. Therefore, the therapeutic role of* Kangfuxin* Solution in severe mucositis needs further investigation.

Data from the trial showed that* Kangfuxin* Solution significantly delayed the time of occurrence of oral mucositis at all levels. The times of occurrence of grades 1, 2, and 3 mucositis were 18.59 ± 8.13 d, 27.98 ± 8.30 d, and 36.88 ± 7.68 d in the test group and 14.48 ± 6.51 d, 23.68 ± 9.22 d, and 30.48 ± 8.84 d in the control group, respectively (*P* < 0.0001, *P* = 0.0014, and *P* = 0.0001, resp.). In addition, the use of* Kangfuxin* Solution allowed the increase of cumulative radiation dose with the emergence of mucositis at grades 1, 2, and 3 (*P* < 0.0001, *P* = 0.0377, and *P* < 0.0001, resp.). Previous studies reported similar results. Peng [35] administered* Kangfuxin* Solution through inhalation to prevent radiotherapy-induced oropharyngeal mucosal reaction in 108 cases in the test group compared with 90 cases in the control group, treated with an anti-inflammatory mouthwash. The results showed that the occurrence of oral mucositis was delayed. Wang et al. [36] used* Kangfuxin* Solution to treat 37 NPC patients with radiation-induced oral mucosa damage. The Vitamin B_12_ and* Kangfuxin* Solution-treated group tolerated a significantly higher radiation dose for the same level of oral mucosal damage. Therefore,* Kangfuxin* Solution increased patients' tolerance to increased radiation dose for similar grade of oral mucositis to enable patient adherence to the complete course of radiotherapy. Therefore, treatment with* Kangfuxin* Solution may affect the prognosis. However, a follow-up study needs to be performed to confirm this hypothesis.

In this trial, VRS was used to evaluate oral pain.* Kangfuxin* Solution reduced the incidence of high-grade oral pain compared with the control group. Oral pain prevents feeding and affects the patients' general physiological condition. It requires additional analgesic treatments, parenteral nutrition, and liquid alternative therapy [7–10]. More importantly, patients often suffer from a psychological burden resulting in higher chances of treatment nonadherence due to subjective factors.* Kangfuxin* Solution reduces oral pain and increases patient compliance.* Kangfuxin* Solution can be either used as a mouth rinse or swallowed. As a secondary efficacy end-point in this study, we also examined whether* Kangfuxin* Solution reduced the incidence of gastrointestinal mucositis. The results showed that* Kangfuxin* Solution reduced the incidence of upper gastrointestinal mucositis in the test group, compared with the control group (*P* < 0.0001), suggesting a potential role in the prevention and treatment of gastrointestinal inflammation. However, this efficacy of Kangfuxin Solution still needs to be confirmed by rigorous clinical trials.

No serious adverse events were observed during the trial. The rate of adverse events, adverse event severity, and outcome were not significantly different between the test and control groups. These data suggest that the safety of* Kangfuxin* Solution is not a concern in clinical practice. Due to the user-friendly approach via mouth rinsing, the patient may generally show a better compliance than other treatments.

The drawbacks of this trial include the following: (1) lack of consensus on the evaluation of efficacy for the treatment of mucositis, due to which the current trial was designed to test for general differences rather than superiority; (2) relatively small sample size and short observation period used in this trial failure to evaluate the long-term toxicity of* Kangfuxin* Solution and the prognosis of NPC patients following treatment; and (3) exclusion of 5-fluorouracil- (5-FU-) treated cases, even though 5-FU is one of the most commonly used chemotherapy drugs for nasopharyngeal carcinoma. The results do not indicate whether* Kangfuxin* Solution can be used in combination with 5-FU to treat patients with nasopharyngeal carcinoma. Additional studies are needed to address the foregoing limitations.

In summary,* Kangfuxin* Solution effectively prevents chemoradiotherapy-induced oral mucositis, reduces the incidence of upper gastrointestinal inflammation, and decreases the severity of oral pain, compared with compound borax gargle. It improves the quality of life in patients. It is effective, user-friendly, safe, and appropriate for clinical application.

## Figures and Tables

**Table 1:** Demographics and baseline characteristics of the efficacy population *N* (%).

Characteristics	Test group	Control group	*P*
Gender			
F	70 (65.4)	83 (76.9)	0.064^*∗*^
M	37 (34.6)	25 (23.2)	
Age	46.3 ± 11.0	48.0 ± 10.0	0.241^*∗∗∗*^
Height (cm)	162.4 ± 7.7	163.4 ± 6.9	0.283^*∗∗∗*^
Weight (kg)	59.3 ± 10.9	61.7 ± 10.5	0.100^*∗∗∗*^
Allergic history			
No	102 (95.3)	105 (97.2)	0.499^*∗∗*^
Yes	5 (4.7)	3 (2.8)	
Clinical stage			
I/II	12 (11.2)	18 (16.7)	0.649^*∗∗*^
III/IV	95 (88.8)	90 (83.3)	
KPS			
100	3 (2.8)	3 (2.8)	0.629^*∗∗*^
90	96 (89.7)	92 (85.2)	
80	8 (7.5)	12 (11.1)	
70	0 (0.00)	1 (0.92)	
TX			
XRT	3 (2.8)	8 (7.4)	0.126^*∗*^
CRT	104 (97.2)	100 (92.6)	
TX time (day)	48.3 ± 37.3	43.6 ± 41.5	0.388^*∗∗∗*^
Medical history			
No	87 (81.3)	85 (78.7)	0.633^*∗*^
Yes	20 (18.7)	23 (21.3)	
Existing history of disease			
No	93 (86.9)	95 (88.0)	0.817^*∗*^
Yes	14 (13.1)	13 (12.0)	

^*∗*^Chi-squared test.

^*∗∗*^Fisher exact probability method.

^*∗∗∗*^ *t*-test.

M, male; F, female; TX, treatment; XRT, radiotherapy; CRT, chemoradiotherapy.

**Table 2:** Comparison of incidence of oral mucositis at the end of trial *N* (%).

Group	G0	G1	G2	G3	*P* ^*∗*^
Test group (107)	5 (4.7)	26 (24.3)	33 (30.8)	43 (40.2)	0.0084
Control group (108)	0 (0.0)	15 (13.9)	35 (32.4)	58 (53.7)	

^*∗*^Rank-sum test.

G, grade.

**Table 3:** Change in oral mucosa grade during the trial *N* (%)^*∗*^.

Group	No change	Reducing 1 grade	*P* ^*∗∗*^
Test group (107)	96 (89.7)	11 (10.3)	0.0098
Control group (108)	106 (98.2)	2 (1.9)	

^*∗*^Change refers to the highest grade of oral mucosa during the treatment subtracted from the oral mucosal grade at the end of treatment.

^*∗∗*^Rank-sum test.

**Table 4:** Time to occurrence of oral mucositis (days).

Grade	Group	Number	Mean	SD	*P* ^*∗*^
G1	Test group	106	18.6	8.1	<0.0001
	Control group	108	14.5	6.5	

G2	Test group	83	28.0	8.3	0.0014
	Control group	95	23.7	9.2	

G3	Test group	43	36.9	7.7	0.0002
	Control group	58	30.5	8.8	

^*∗*^ *t*-test.

SD, standard deviation.

**Table 5:** Cumulative radiation dose of occurrence of oral mucositis (Gy).

Grade	Group	Number	Mean	SD	*P* ^*∗*^
G1	Test group	106	27.9	11.0	<0.0001
	Control group	108	22.1	8.9	

G2	Test group	82	42.0	11.0	0.0377
	Control group	95	37.3	18.7	

G3	Test group	43	56.2	10.2	<0.0001
	Control group	58	46.0	12.1	

^*∗*^ *t*-test.

SD, standard deviation.

**Table 6:** Incidence of upper gastrointestinal mucositis and oral pain.

Group	G0	G1	G2	G3	*P* ^*∗*^
Upper gastrointestinal mucositis					
Test group (107)	33 (30.8)	36 (33.6)	35 (32.7)	3 (2.8)	<0.0001
Control group (108)	9 (8.3)	36 (33.3)	58 (53.7)	5 (4.6)	
Oral pain					
Test group (107)	12 (11.2)	43 (40.2)	49 (45.8)	3 (2.8)	0.0003
Control group (108)	1 (0.9)	30 (27.8)	73 (67.6)	4 (3.7)	

^*∗*^Rank-sum test.
